# Correction: Proper migration of lymphatic endothelial cells requires survival and guidance cues from arterial mural cells

**DOI:** 10.7554/eLife.90048

**Published:** 2023-06-12

**Authors:** Di Peng, Koji Ando, Melina Hußmann, Marleen Gloger, Renae Skoczylas, Naoki Mochizuki, Christer Betsholtz, Shigetomo Fukuhara, Stefan Schulte-Merker, Nathan D Lawson, Katarzyna Koltowska

**Keywords:** Zebrafish

Peng D, Ando K, Hußmann M, Gloger M, Skoczylas R, Mochizuki N, Betsholtz C, Fukuhara S, Schulte-Merker S, Lawson ND, Koltowska K. 2022. Proper migration of lymphatic endothelial cells requires survival and guidance cues from arterial mural cells. *eLife* **11**:e74094. doi: 10.7554/eLife.74094.

Published 22 March 2022

This error occurred during the misaligned “copy and paste” from the primer list while drafting the paper. It did not affect anyone qPCR experiments or the results. We regret any confusion or difficulties it may have caused.

In the Materials and methods session - FACS and qPCR analysis, there is a mix-up for the dll4 primers

Corrected sequence:

GGACAAATGCACCAGTATGC

GTTTGCGCAGTCGTTAATGT

Original sequence:

CGTTCCCAGGAGCCTTTTCT

TTGGGATCAGGGATGGGGAT

The article has been corrected accordingly.

